# AI-assisted diagnosis of vulvovaginal candidiasis using cascaded neural networks

**DOI:** 10.1128/spectrum.01691-24

**Published:** 2024-11-22

**Authors:** Zhongxiao Wang, Ruliang Wang, Haichun Guo, Qiannan Zhao, Huijun Ren, Jumin Niu, Ying Wang, Wei Wu, Bingbing Liang, Xin Yi, Xiaolei Zhang, Shiqi Xu, Xianling Dong, Liqun Wang, Qinping Liao

**Affiliations:** 1 Hebei Key Laboratory of Nerve Injury and Repair, Chengde Medical University, Chengde, China; 2 Department of Obstetrics and Gynecology, Beijing Tsinghua Changgung Hospital, School of Clinical Medicine, Tsinghua University, Beijing, China; 3 Changsha Hospital for Maternal & Child Health Care, Changsha, China; 4 Department of Clinical Laboratory, Yantaishan Hospital, Yantai City, China; 5 Department of Clinical Laboratory, The First Affiliated Hospital of Zhengzhou University, Zhengzhou, China; 6 Shenyang Women’s and Children’s Hospital, Shenyang, China; 7 Beijing Turing Medlab Co., Ltd., Beijing, China; 8 Hebei International Research Center of Medical Engineering, Chengde Medical University, Chengde, China; 9 Jiangxi Maternal & Child Health Hospital, Nanchang, China; Memorial Sloan Kettering Cancer Center, New York, New York, USA

**Keywords:** Vulvovaginal candidiasis, cascaded neural network, slide level, microscopic images

## Abstract

Vulvovaginal candidiasis (VVC) is a prevalent fungal ailment affecting women globally. Timely and accurate diagnosis is crucial. Traditional methods, relying on clinical evaluation and manual microscopic examination, have limitations. Artificial intelligence (AI) offers potential improvements in diagnostic accuracy and efficiency by objectively analyzing microscopic images of lower genital tract infections in women. A cascaded model was developed using 100,387 microscope images and 1,761 slides to diagnose VVC at slide level. The model’s diagnostic accuracy was compared with experts’. Five hundred thirteen slides were used to evaluate whether the experts’ diagnostic skills could be improved using the model as an AI-assisted tool. The consistency between experts’ interpretations of microscopic digital images and microscopic examination under eyepiece was assessed to determine whether the collected images adequately represented the slides. The model obtained AUC = 0.9447, 0.9711, and 0.9793 for slide-level diagnosing yeast hyphae, budding yeast, and yeast. Compared with the average performance of experts, the Youden indexes of our model’s best points were improved by 0.0069, 0.0772, 0.0579, and 0.0907 for yeast hyphae, budding yeast, yeast, and VVC. The average accuracy of the experts was improved by 5.98%, 5.20%, 4.82%, and 8.19% using our model as an AI-assisted tool. The consistency rates and Cohen’s kappa coefficients between experts’ interpretations of microscopic digital images and microscopic examination under eyepiece exceeded 93% and 0.83 for the three different morphologic states of yeast. Our model exhibits superior diagnostic accuracy for VVC compared to experts. Experts can significantly improve their own diagnostic accuracies by using our model as an AI-assisted tool. The microscope images collected from each slide effectively represent the slide.

**IMPORTANCE:**

A cascaded deep neural network model was developed for slide-level diagnosis of vulvovaginal candidiasis (VVC), demonstrating superior diagnostic accuracy compared to experts. Experts significantly enhanced their diagnostic accuracies by utilizing our model as an AI-assisted tool. Therefore, this model holds potential for clinical application to aid in the diagnosis of VVC.

## INTRODUCTION

Vulvovaginal candidiasis (VVC), more commonly known as a yeast infection, is a prevalent fungal infection that affects women worldwide. This condition is characterized by distressing symptoms such as vaginal itching, a burning sensation, unusual discharge, and discomfort during sexual intercourse, typically caused by the Candida species, notably Candida albicans. VVC affects approximately 70%–75% of women during their lifetime, making it the second most common cause of vaginitis symptoms after bacterial vaginosis (BV) (1–3).

Accurate and timely diagnosis of VVC is paramount for effective treatment and management. The conventional approach to diagnosing VVC relies on clinical assessment, patient history, and microscopic examination of vaginal swabs or discharge samples. Microscopic examination plays a pivotal role in detecting the presence of pathogenic microorganisms, including yeast pseudohyphae, budding yeast, and yeast. Yeast refers to the single-cell yeast morphology which are the reproductive cells of fungi, typically characterized by their small, round to oval shape. Yeast can exist in the vagina of healthy women, so the presence of yeast in the vaginal discharge is not a sign of VVC. Budding yeast typically appear as oval or round cells with smaller buds attached, often observed in various stages of division. Pseudohyphae are elongated, budding yeast cells that remain attached after division, forming chain-like structures. They appear as elongated cells with constrictions at the points of septation. The pathological process of vulvovaginal candidiasis (VVC) is primarily divided into three stages: asymptomatic colonization, the transition from the yeast (non- budding) phase to the budding yeast phase, and the invasion of the vaginal epithelium by the pseudohyphal phase. For proper diagnosis, the presence of (pseudo-)hyphae or budding yeast is always necessary which is the clinical criteria of diagnosing VVC (4). Although asymptomatic colonization during the yeast phase is diagnosed as VVC-negative in laboratory tests, the presence of Candida yeasts on the vaginal mucosa is a necessary condition for the development of VVC. The detection and reporting of colonizing yeasts, in contrast to reports where no yeasts are identified, may suggest a relatively higher risk of VVC development and hold significant clinical value in indicating the potential risk of VVC. Therefore, our model also performed diagnostic recognition of yeast. Common diagnostic methods including 10% potassium hydroxide preparation and gram staining demonstrate comparable sensitivity and specificity for diagnosing VVC alone (1, 3). However, given the coexistence of VVC and BV leading to mixed infections, gram staining of vaginal smears becomes an optimal method for identifying VVC and potential mixed bacterial infections (5, 6).

Nonetheless, the accuracy and consistency of VVC diagnosis based on microscopic examination, particularly through gram-staining smears, can vary among healthcare professionals. Interpretation of microscopic images demands a high level of expertise and experience, and errors in interpretation or subjective differences can lead to diagnostic inaccuracies. Moreover, the manual examination process is time-intensive, laborious, and susceptible to human errors, thereby limiting its efficiency and reliability. To address these challenges, there is a growing interest in leveraging artificial intelligence (AI) and computer-aided diagnostic systems for diagnosing lower genital tract infections in females. AI models can analyze microscopic images and offer automated, objective assessments, potentially enhancing diagnostic accuracy and efficiency.

In recent years, a series of Convolutional Neural Network (CNN) models have been proposed to enhance image recognition capabilities (7–11). These models have surpassed human performance in tasks such as image classification, image semantic segmentation, and image object detection. Consequently, numerous CNN models have been developed for processing medical images, demonstrating excellent performance (12–18). The diagnosis of VVC requires identifying yeast pseudohyphae, budding yeast, and yeast from microscope images, necessitating the use of object detection CNN models. R-CNN series and YOLO series CNN models have achieved notable success in target detection tasks in recent years (19–27). In this paper, the YOLOv5 model (27) was employed for image-level diagnosis.

Few studies have focused on the automated diagnosis of VVC in recent years, and the limited existing research has notable shortcomings: (28, 29) (i) previous studies concentrated solely on image-level diagnosis. The diagnosis process relied on traditional machine-learning algorithms, and the process was complicated. (ii) Only a small data set of a few hundred microscope images was utilized for training and testing. (iii) Data collection was restricted to a single medical center, posing challenges in verifying the model’s generalization ability. (iv) No comparative experiments were conducted to assess the diagnostic abilities of both humans and the model. (v) The potential enhancement of human diagnostic abilities through the model was not investigated. To address these issues, our research implemented the following improvements: (i) a cascaded model was devised for slide-level VVC diagnosis. This model comprises a YOLO model for image-level VVC detection and a three-layer fully connected (FC) neural network for predicting the diagnosis result at the slide level. This end-to-end model simplifies the inference process. (ii) We collected a comprehensive data set of 100,387 microscope images and 1,761 slides from four hospitals to train and evaluate the cascaded model. (iii) Comparative analysis with experts demonstrated that the cascaded model exhibited diagnostic abilities close to or even better than those of experts. (iv) Experts significantly enhanced their diagnostic skills when utilizing the model as an AI-assisted tool. Moreover, our methodology standardized the process of collecting images from slides, and an evaluation was conducted to ensure the representativeness of the collected images for each slide.

## MATERIALS AND METHODS

### Data preparation

#### Data collection

A total of 123 vaginal discharge slides from patients treated at Changsha Hospital for Maternal & Child Health Care between April 2022 and December 2022 were gathered to train the YOLO (image-level) model. Vaginal discharge swabs were obtained from the posterior fornix by physicians using Harvey Psw-1 (Suzhou Turing Microbial Technology Co., Ltd.) and stored in a preservation solution. The slides preparation and Gram-staining were then completed in the liquid-based cytology slide-prep machine (Koch SG15, Suzhou Turing Microbial Technology Co., Ltd). Then, the slides were imaged using an automatic microscope scanner (Abbe AutoScan X4, Suzhou Turing Microbial Technology Co., Ltd). Subsequently, 476 additional patient slides from April 2022 to December 2022 at Changsha Hospital for Maternal & Child Health Care and the First Affiliated Hospital of Zhengzhou University were collected for testing the YOLO model.

For the development of the cascaded (slide-level) model, a combined data set of 1,761 vaginal discharge slides from patients treated at Changsha Hospital for Maternal & Child Health Care, Yantaishan Hospital, Jiangxi Maternal & Child Health Hospital, and the First Affiliated Hospital of Zhengzhou University, spanning April 2022 to April 2023, was utilized. Among these, 1,248 samples were randomly assigned to the training set, while the remaining 513 samples were constituted the test set.

Our study was conducted in accordance with the Declaration of Helsinki (as revised in 2013) and was approved by the ethics committees of participating hospitals, which waived the requirement of informed consent for this retrospective analysis.

#### Image-level data

A total of 100,387 microscopic images extracted from 599 slides were compiled to construct an image-level object detection model. Sample collection and images generation were the same as described in Data collection. Each image covered an actual physical area of 226.9 µm × 142.5 µm, with a resolution of 1,936 × 1,216 pixels.

The distribution of samples at the image level is detailed in Table 1. For the training set, 5,435 images from 123 slides (all sourced from VVC positive patients) were chosen. This set comprised 1,715 yeast hyphae-positive images, 1,954 budding yeast-positive images, and 4,800 yeast-positive images. The remaining 94,952 images, gathered from 476 slides (107 slides from VVC positive patients and 369 slides from VVC negative patients), constituted the test set. This set encompassed 1,101 yeast hyphae-positive images, 4,152 budding yeast-positive images, and 7,956 yeast-positive images. Certain images exhibited more than two types of targets. One hundred two images in the training set did not contain any yeast morphologies. It is notable that the proportion of positive images in the test set did not exceed 10% due to the minimal presence of positive images in clinical data.

**TABLE 1:** The distribution of the image-level data set

	Training set			Testing set			Total		
	Positive	Negative	Total	Positive	Negative	Total	Positive	Negative	Total
Yeast hyphae	1,715	3,720	5,435	1,101	93,851	94,952	2,816	97,571	100,387
Budding yeast	1,954	3,481	5,435	4,152	90,800	94,952	6,106	94,281	100,387
Yeast	4,800	635	5,435	7,956	86,996	94,952	12,756	87,631	100,387

**Fig 1:**
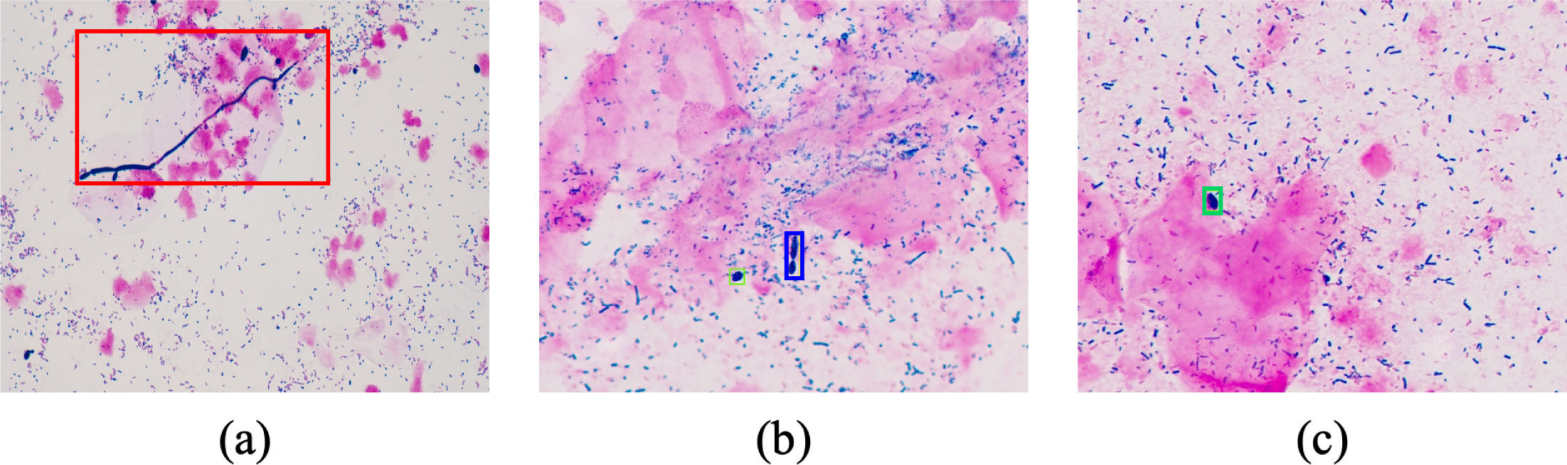
The typical images of three different morphologic states of yeast. (a) was a typical image of yeast hyphae (in the red box). (b) was a typical image of budding yeast (in the blue box). (c) was a typical image of a yeast (in the green box).

All positive samples at the image level were labeled by experts affiliated with Beijing Turing Medlab Co., Ltd., Beijing Tsinghua Changgung Hospital, and Yantaishan Hospital. All experts involved in label work have the qualification certificate of clinical laboratory technician in detecting yeast hyphae, budding yeast, and yeast. They have undergone at least 1 year of training and possess 2 years of clinical laboratory experience in interpreting clinical vaginal smears. Each sample underwent labeling by three microbiologists, with the consistent results considered the ground truth. In cases of inconsistent annotations, the respective image was excluded from further analysis. Figure 1 illustrates typical instances of labeled samples: Fig. 1a depicts a labeled yeast hyphae sample, Fig. 1b represents a labeled budding yeast sample, and Fig. 1c shows a labeled yeast sample.

#### Slide-level data

A total of 1,761 slides were collected for the development of the cascaded model. Each slide contained a grid of 5 rows × 40 columns of images, as depicted in Fig. S1. The distribution of slide-level samples was presented in Table 2. Among these, 1,248 slides were designated as the training set, comprising 303 yeast hyphae-positive slides, 491 budding yeast-positive slides, 513 yeast-positive slides, and 530 VVC-positive slides. The presence of yeast hyphae or budding yeast was interpreted as VVC-positive, but the absence of any yeast morphologies or the presence of only yeast was interpreted as VVC-negative. The remaining 513 slides constituted the test set, which included 120 yeast hyphae-positive slides, 188 budding yeast-positive slides, 205 yeast-positive slides, and 200 VVC-positive slides. It should be noted that certain slides contained more than two types of targets.

**TABLE 2:** The distribution of the slide-level data set

	Training set			Testing set			Total		
	Positive	Negative	Total	Positive	Negative	Total	Positive	Negative	Total
Yeast hyphae	303	945	1,248	120	393	513	423	1,338	1,761
Budding yeast	491	757	1,248	188	325	513	679	1,082	1,761
Yeast	513	735	1,248	205	308	513	718	1,043	1,761
VVC	530	718	1,248	200	313	513	730	1,031	1,761

All slides underwent labeling by three experts with a minimum of 5 years of clinical laboratory experience in reading clinical vaginal smears and continue to do so as part of their clinical diagnostic responsibilities, hailing from Yantaishan Hospital, Shenyang Women’s and Children’s Hospital, and the First Affiliated Hospital of Zhengzhou University. Each expert individually scanned the slides under a microscope and provided their diagnostic results, which included identifying the positive or negative of yeast hyphae, budding yeast, and yeast. The diagnostic results of VVC can be inferred from the diagnostic results of yeast hyphae and budding yeast. Consistent results among two or more experts were considered the ground truth. Samples with conflicting results among the three experts were subsequently removed from the data set.

### Development of cascaded deep neural networks model

#### Target detection CNN model for image-level diagnosis

For the morphological automatic diagnosis of VVC, the first step involved detecting three different morphologic states of yeast at the image level. In recent years, a series of object detection models have been developed, with the R-CNN and YOLO series models being the most commonly employed. In this study, we utilized the YOLOv5s model to perform the detection of pathogenic microorganisms at the image level. The model provides outputs indicating the position, size, and positive probability of each target, including the three different morphologic states of yeast. To train the model, three NVIDIA RTX 3090 GPUs were utilized. During the training process, a batch size of 36 was employed, the SGD optimizer was used, the learning rate was 0.01, and the delay was 0.9 per epoch. The loss function incorporated class loss, objectness loss, and location loss. The best-performing model was obtained after training for 36 h and completing 2,000 iterations.

#### Cascade model for slide-level diagnosis

**Fig 2:**
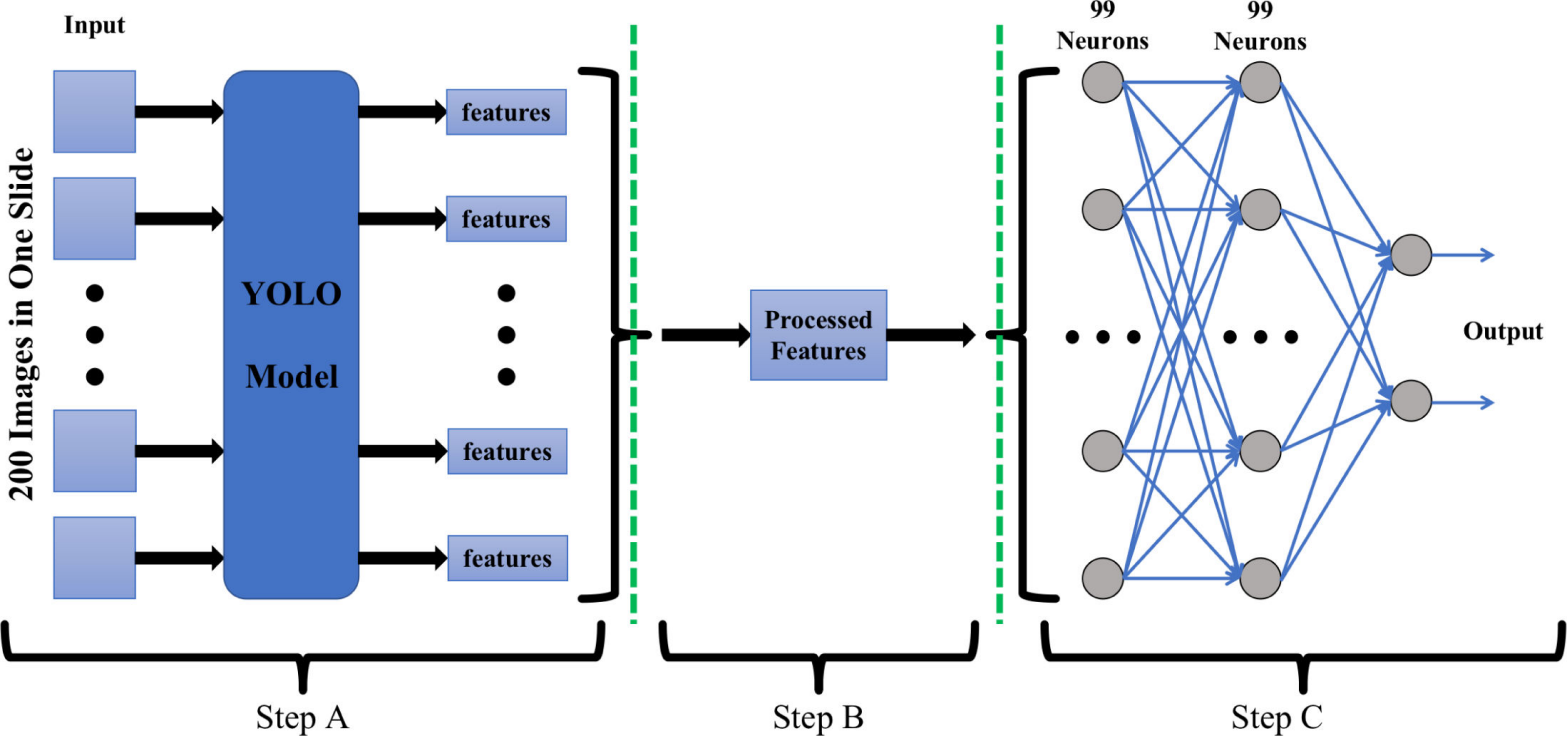
The developed cascade model for slide-level diagnosis involved multiple steps. In Step A, a YOLO model was utilized to detect pathogenic microorganisms in images. This process generated features such as the position, size, and positive probability of each target. Step B involved merging all the extracted features from the 200 images obtained from a single slide and converting them into a 99 elements feature vector. Figure 3 illustrates the conversion of these features into the feature vector. Step C consisted of a three-layer fully connected neural network that received the 99 elements feature vector as input and produced the slide’s positive probability of the pathogenic microorganism.

The YOLO model exclusively provides image-level diagnostic results, while clinical diagnosis necessitates slide-level diagnostic outcomes. Hence, a cascaded model, illustrated in Fig. 2, was devised for slide-level diagnosis.

Within the inference pipeline, the model encompassed three sequential steps—A, B, and C. In Step A, a YOLO model was employed to identify targets in each of the 200 images constituting a slide (Fig. S1 illustrates the process of capturing 200 images from one slide). The YOLO model generated feature sets for each image, comprising details such as the position, size, and positive probability of each target. All image feature sets were then fed into Step B.

**Fig 3:**
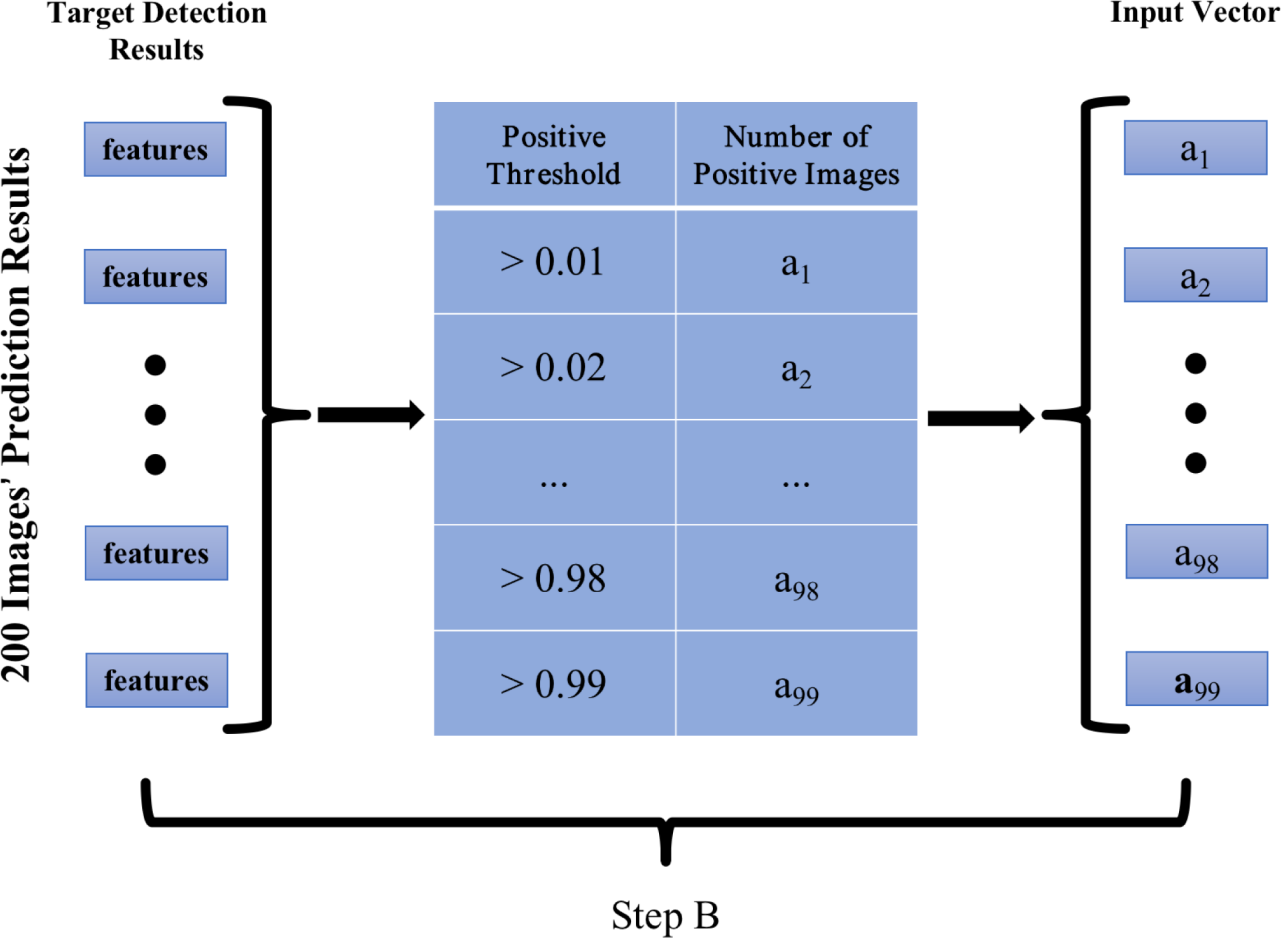
Converted all the features into a feature vector with 99 elements. In the conversion process, 99 positive probability thresholds (ranging from 0.01 to 0.99) were set, if an image had more than one target’s positive probability greater than the positive probability threshold, the image was recorded as a positive sample, the number of positive images was obtained for each threshold, therefore a feature vector with 99 elements corresponding to 99 thresholds was obtained.

Step B, depicted in Fig. 3, involved setting 99 positive probability thresholds (0.01, 0.02, ..., 0.99). If an image exhibited positive probabilities surpassing the threshold for more than one target, it was recorded as a positive sample. The count of positive images was determined for each threshold, resulting in a feature vector with 99 elements corresponding to the 99 thresholds. Subsequently, this feature vector was input into Step C, a three-layer fully connected (FC) neural network. The input and hidden layers each comprised 99 neurons, while the output layer contained two neurons—one for positive probability and another for negative probability of the slide.

During the training process, the YOLO model in Step A and the three-layer FC model in Step C underwent independent training. Initially, the YOLO model was trained using image-level data. Subsequently, the three-layer FC model was trained utilizing slide-level data, where the input comprised the feature vector of each slide from Step B, and the output represented the slide’s positive probability for each of the three different morphologic states of yeast. The optimal positive thresholds (obtained by maximizing the Youden index) for yeast hyphae and budding yeast diagnosis are chosen to ascertain the positivity or negativity of each slide prediction and subsequently to determine the VVC positivity or negativity of each specimen.

### Comparison to microscopists

Three independent healthcare providers (HCPs) were enlisted for the prognosis prediction of slide-level results. These HCPs originated from distinct medical institutions, namely, Changsha Hospital for Maternal & Child Health Care, Shenyang Women’s and Children’s Hospital, and the First Affiliated Hospital of Zhengzhou University.

Each human reader furnished three types of slide-level prediction results. First, the human readers exclusively examined the 200 images collected on each slide and provided their slide-level predictions. Second, the human readers conducted a comprehensive examination of each slide under the microscope and rendered their diagnostic results. Third, the human readers offered predictions by combining image examination with the use of our model as an AI-assisted tool, where the model provides positive or negative instances of yeast hyphae, budding yeast, yeast, and VVC, while also providing three images corresponding to three different morphological states of yeast with the highest positive probabilities. The three prediction methods are spaced 1 month apart, and the sequence of specimens has been deliberately disrupted each time to prevent the influence of previous predictions on subsequent ones, thereby mitigating any potential memory-related biases.

The evaluation involved three types of comparison results, utilizing the outcomes from our cascade model and the three human readers. First, the results from human readers who examined the 200 digital images and conducted microscopic examinations under the eyepiece were compared to ascertain whether the 200 microscope digital images collected from each slide adequately represented the slide. Second, the prediction accuracy between our cascade model and the three human readers who exclusively examined images was compared to determine if our model exhibited superior performance compared to human readers. Third, the performance of human readers examining the digital images only and those utilizing our model as an AI-assistant tool were compared to assess whether our model enhanced the overall performance of human readers. The metric methods employed for comparing the results of these comparisons are delineated below.

### Analysis of diagnostic performance using metrics methods

The consistency rate and Cohen’s kappa coefficient were employed to evaluate the agreement among human readers who examined the 200 images and scanned the slide. Sensitivity and specificity were utilized to assess the performance of human readers. Since our model exclusively provided positive probabilities for the three different morphologic states of yeast per sample, it was more appropriate to employ AUC (Area Under the Curve) and ROC (Receiver Operating Characteristic) curves to illustrate the performance of our model. The Youden index was used to determine the optimal threshold point on the ROC curve. Accuracy was employed to quantify the degree of improvement achieved by human readers utilizing our model as an AI-assisted tool.

## RESULTS

### The consistency of human reader between only reading images and only scanning slides

To assess the representativeness of the 200 microscope images collected from each slide, the consistency of each human reader between solely reading images and exclusively scanning slides was evaluated. Table 3 illustrates the consistency rates between human readers’ prediction results based on digital images reading only and microscopic examination under eyepiece only. The consistency rates were calculated by dividing the number of agreed specimens by the total number of specimens. Additionally, Cohen’s kappa coefficients were also calculated to assess the agreement rate. The results show all consistency rates for the three different morphologic states of yeast exceeding 93%, and all kappa coefficients surpassing 0.83. These results indicate that the human readers achieved sufficiently high consistency between image examination and slide scanning. Consequently, it can be inferred that the 200 microscope images collected from each slide effectively represent the slide.

**TABLE 3:** The consistency rates of human readers between reading digital images and microscopic examination under eyepiece

	Consistency rate			Kappa		
	Yeast hyphae	Budding yeast	Yeast	Yeast hyphae	Budding yeast	Yeast
Expert1	94.54%	96.10%	97.27%	0.8558	0.9194	0.9434
Expert2	93.76%	96.69%	98.25%	0.8322	0.9286	0.9633
Expert3	95.91%	95.91%	96.49%	0.8841	0.9127	0.9266

### The performance of image-level model

The image-level model employed in this study utilized the YOLOv5s model, which provided outputs indicating the position, size, and positive probability of each target. In clinical practice, the primary concern is whether the target can be detected in the image. Therefore, the positive probability of the target in each image was considered. During the testing phase, the positive probability of the target with the highest value served as the overall positive probability for that particular image. Consequently, each image was assigned three positive probabilities corresponding to the three types of microbes.

For the evaluation of model performance, the AUC (Area Under the Curve) metric was employed. The obtained results for yeast hyphae yielded an AUC of 0.8778, while budding yeast demonstrated an AUC of 0.9666, and yeast presented an AUC of 0.9829. These values indicate the efficacy of the model in accurately predicting the presence of each respective type of microbe.

### The performance of slide-level model

The slide-level model in this study was constructed by cascading a YOLO model and a three-layer fully connected neural network. The model’s outputs consisted of the positive probabilities associated with yeast hyphae, budding yeast, and yeast at the slide level. The diagnostic outcome for VVC, whether positive or negative, can be determined by selecting the optimal positive thresholds for yeast hyphae and budding yeast. For testing the slide-level model, a total of 513 slides were collected as the test set (further details can be found in Table 2).

**Fig 4:**
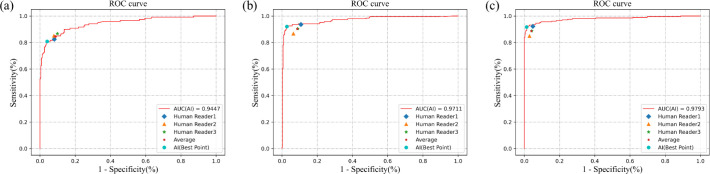
The performance of our cascaded model and three human practitioners in the slide-level diagnosis. (a) was the performance on yeast hyphae diagnosis, (b) was the performance on budding yeast diagnosis, and (c) was the performance on yeast diagnosis. The model demonstrated superior performance compared to the average performance of the three human practitioners in diagnosing budding yeast and yeast. Additionally, the model’s diagnostic performance in yeast hyphae was comparable to the average performance of the three human practitioners.

The performance of the slide-level model on the test set is illustrated in Fig. 4. The model achieved an AUC of 0.9447 for yeast hyphae, 0.9711 for budding yeast, and 0.9793 for yeast detection. At the optimal threshold point on the ROC curve, the Youden indexes were determined: 0.7676 for yeast hyphae (with sensitivity and specificity values of 0.8083 and 0.9593, respectively), 0.8925 for budding yeast (with sensitivity and specificity values of 0.9202 and 0.9723, respectively), 0.9041 for yeast (with sensitivity and specificity values of 0.9171 and 0.9870, respectively), and 0.8939 for VVC (with sensitivity and specificity values of 0.9450 and 0.9489, respectively). These results demonstrate the slide-level model’s effectiveness in predicting the presence of different pathogenic microorganisms. To further illustrate the erosion of the model’s sensitivity in detecting true cases, the P-R curves are presented in Fig. S2.

### Performance comparison between cascaded model and human practitioners on slides

The performance of the cascaded model (slide-level model) demonstrated comparability with that of three experts, all hailing from esteemed medical institutions in China, each possessing more than 5 years of clinical experience. Figure 4 illustrates the performance of our model and the three experts, while Table 4 provides detailed information on the performance of the experts and our AI model with optimal positive probability threshold points (corresponding to the point on the ROC curve that maximizes the Youden index), where the groud truth was described in Slide-level data.

**TABLE 4:** The performance of human readers and the best point of our model

	Sensitivity				Specificity				Youden index			
	Yeast hyphae	Budding yeast	Yeast	VVC	Yeast hyphae	Budding yeast	Yeast	VVC	Yeast hyphae	Budding yeast	Yeast	VVC
Expert1	82.50%	93.62%	92.20%	95.50%	91.86%	89.23%	95.13%	85.62%	0.7436	0.8285	0.8733	0.8112
Expert2	85.00%	86.70%	84.88%	97.00%	92.11%	93.54%	97.08%	82.43%	0.7711	0.8024	0.8196	0.7943
Expert3	86.67%	90.43%	88.78%	93.50%	90.08%	91.08%	95.78%	86.90%	0.7675	0.8151	0.8456	0.8040
Average	84.72%	90.25%	88.62%	95.33%	91.35%	91.28%	96.00%	84.98%	0.7607	0.8153	0.8462	0.8032
AI	80.83%	92.02%	91.71%	94.50%	95.93%	97.23%	98.70%	94.89%	0.7676	0.8925	0.9041	0.8939

The experts, on average, exhibited sensitivities of 84.72%, 90.25%, 88.62%, and 95.33% for yeast hyphae, budding yeast, yeast, and VVC, respectively, with corresponding average specificities of 91.35%, 91.28%, 96.00%, and 84.98%. The average Youden indexes were calculated as 0.7607, 0.8153, 0.8462, and 0.8032. Notably, the optimal points of our model displayed superior performance compared to the experts’ average levels, with higher Youden indexes of 0.0069, 0.0772, 0.0579, and 0.0907 for yeast hyphae, budding yeast, yeast, and VVC, respectively. With the exception of the sensitivity of yeast hyphae, all other sensitivities and specificities either reached or surpassed the average levels achieved by the experts. For a more detailed comparison, the quantity of false negative and false positive samples with AI-alone prediction and human-alone prediction are presented in Table S1.

### Improvement of human practitioners’ performance with cascaded model

The impact of our model on the diagnostic accuracy of human practitioners was also investigated. Table 5 clearly demonstrates that our model significantly enhanced the diagnostic accuracy of human practitioners. The accuracy for each morphologic state of yeast, across all human practitioners, witnessed an improvement ranging from 3.90% to 6.66%. On average, the overall accuracy increased by 5.98%, 5.20%, 4.82%, and 8.19% for yeast hyphae, budding yeast, yeast, and VVC, respectively. Thus, as an AI-assisted tool, our model made a substantial improvement in the diagnostic accuracy of the experts. To illustrate the details of accuracy improvement, the quantity of false negative and false positive samples with human alone prediction and human + AI prediction are presented in Table S1.

**TABLE 5:** The effectiveness of improving the diagnostic accuracy of human readers by employing the cascaded model as AI-assisted tool

	Only human readers				Using AI-assisted				Improvement			
	Yeast hyphae	Budding yeast	Yeast	VVC	Yeast hyphae	Budding yeast	Yeast	VVC	Yeast hyphae	Budding yeast	Yeast	VVC
Expert1	89.67%	90.84%	93.96%	89.47%	96.30%	95.91%	97.86%	97.66%	6.63%	5.07%	3.90%	8.19%
Expert2	90.45%	91.03%	92.20%	88.11%	95.13%	95.71%	97.86%	96.69%	4.68%	4.68%	5.66%	8.58%
Expert3	89.28%	90.84%	92.98%	89.67%	95.91%	96.69%	97.86%	97.47%	6.63%	5.85%	4.88%	7.80%
Average	89.80%	90.90%	93.04%	89.02%	95.78%	96.10%	97.86%	97.27%	5.98%	5.20%	4.82%	8.19%

## DISCUSSION

Current studies on the automatic diagnosis of VVC predominantly concentrate on image-level diagnosis, with limited attention given to slide-level diagnosis, posing challenges for clinical application. Additionally, these studies exclusively employ traditional machine-learning algorithms, involving a complex process encompassing preprocessing, segmentation, feature extraction, and classification. In contrast, our study introduced two enhancements to streamline the diagnostic approach: (i) in response to clinical needs, our study provides both image-level and slide-level diagnosis results, establishing a robust foundation for the clinical implementation of automatic VVC diagnosis. (ii) Our study directly employed deep learning technology’s object detection algorithms for end-to-end prediction diagnosis, simplifying the intricate procedural steps. Furthermore, scant attention has been given to the image capture process from slides in previous research. In this study, we delve into the methodology of capturing images from slides and validate the collected images’ ability to faithfully represent the entire slide, thereby standardizing the image capture process. Through a comparative analysis of expert evaluations between image readings and slide scans, we observed a noteworthy level of agreement, signifying that the 200 images collected from each slide sufficiently encapsulate the slide’s content.

In recent years, the CNN model has demonstrated increasingly superior performance in object detection tasks, driven by advancements in deep learning technology. Among the various object detection models, the R-CNN series and YOLO series models have gained prominence. In this study, which involves typical object detection tasks at the image level, YOLO was employed to detect pathogenic microorganisms in image-level samples. The test results revealed that the YOLOv5s model exhibited better performance in detecting the three different morphologic states of yeast at the image level. For clinical diagnosis, slide-level diagnostic results were required. To address this, a cascaded model was constructed, merging the outputs of the image model to generate slide-level results. In our cascaded model, we explored five merging methods, which included (i) taking the output positive probability of the image with the maximum positive probability of the detected target as the slide’s output. (ii) For each image, extracting the top-*k* (*k* = 1,2,3,4) maximum positive probabilities of the detected target, and concatenating the 200*k positive probabilities into a one-dimensional vector. (iii) Concatenating all positive probabilities of the detected targets across all 200 images into a one-dimensional vector. (iv) Concatenating all positive probabilities and the size of the detected target across all 200 images into a one-dimensional vector. (v) The merging method employed in this study. The test results showed that the merging method utilized in this research yielded better performance for the cascaded model.

Our cascaded model exhibited strong performance in diagnosing the three different morphologic states of yeast, with all AUCs surpassing 0.94. In comparison to the average level of experts, the model demonstrated superior performance in diagnosing budding yeast and yeast. Specifically, the model’s best points exhibited higher sensitivities by 1.77% and 3.09%, along with higher specificities by 5.95% and 2.70%, respectively. For yeast hyphae diagnosis, the specificity exceeded the average level of experts by 4.58%, but the sensitivity was 3.89% lower. This discrepancy can be attributed to the relatively typical morphology of budding yeast and yeast observed in microscope images, whereas yeast hyphae morphology proves to be more complex and diverse. Consequently, the model’s sensitivity for yeast hyphae diagnosis was lower compared to that of budding yeast and yeast. However, by augmenting the training set with additional yeast hyphae samples encompassing a wider range of morphologies, the model’s diagnostic ability for yeast hyphae could be enhanced. We utilized three NVIDIA GeForce RTX 3090 graphics processing units (GPUs) for training the YOLO model and the slide-level model. An NVIDIA GeForce RTX 2070 SUPER GPU was utilized in the inference process. The entire prediction process, from slide scanning to result prediction using the cascaded model, took a total of 3 min.

Experts can notably enhance their diagnostic proficiency by leveraging our model. The diagnostic accuracies of all three experts for the three different morphologic states of yeast were augmented by over 3.9%. Additionally, the average diagnostic accuracies of these experts improved by more than 4.8% with the aid of our model. Thus, our model serves as a valuable auxiliary tool to assist human experts in diagnosing VVC. Our model provided binary classification results for yeast hyphae, budding yeast, yeast, and VVC. During the diagnostic process, when our model was employed as an auxiliary model, the specimans with inconsistent diagnosis results between the model and the expert garnered the attention of the expert. If the expert’s diagnosis of these specimens was incorrect without AI assistance, the model’s reminder could rectify the diagnosis. Therefore, our model can improve the expert’s performance.

Our model demonstrated commendable performance in diagnosing VVC. However, our study does have several limitations that should be acknowledged. First, the sensitivity of yeast hyphae in our model’s best point did not surpass the average level of experts, indicating the need for further improvement in the model’s performance for yeast hyphae diagnosis. Second, the training and testing data utilized in our model were limited to four hospitals. To ascertain the generalization ability of our model, it is crucial to collect additional data from a more diverse range of medical centers. Lastly, the man-machine comparison in our study involved only three experts. To obtain a comprehensive evaluation, it is recommended to include more experts with varying diagnostic abilities in future man-machine comparisons.

In summary, our study pioneered the development of a cascaded deep neural network model for the automatic diagnosis of VVC at the slide level, exhibiting superior performance compared to human readers and markedly enhancing the diagnostic skills of experts. Additionally, our research introduced a standardized methodology for capturing representative images from slides. The AI-assisted analysis of Gram-stained microscopic images of vaginal secretions not only elevates the diagnostic accuracy and efficiency of VVC but also broadens its applicability to the detection of various other types of female lower genital tract infections. This innovative approach substantially advances the clinical diagnosis and treatment effectiveness for common vaginal infectious diseases.

## Data Availability

The data that support the findings of this study are available from the corresponding author upon reasonable request.

## SUPPLEMENTAL MATERIAL

Supplemental materials spectrum.01691-24-s0001.docx Fig. S1 and S2; Table S1.

ASM does not own the copyrights to Supplemental Material that may be linked to, or accessed through, an article. The authors have granted ASM a non-exclusive, world-wide license to publish the Supplemental Material files. Please contact the corresponding author directly for reuse.
