# Supplementary material for: AI-assisted diagnosis of vulvovaginal candidiasis using cascaded neural networks
Source: Microbiol Spectr. 2024 Nov 22;13(1):e01691-24. doi: 10.1128/spectrum.01691-24 (PMC11705804; doi:10.1128/spectrum.01691-24)
Supplement: Supplemental materials — Fig. S1 and S2; Table S1. [file spectrum.01691-24-s0001.docx]

**Supplemental Materials.**

**Figures**


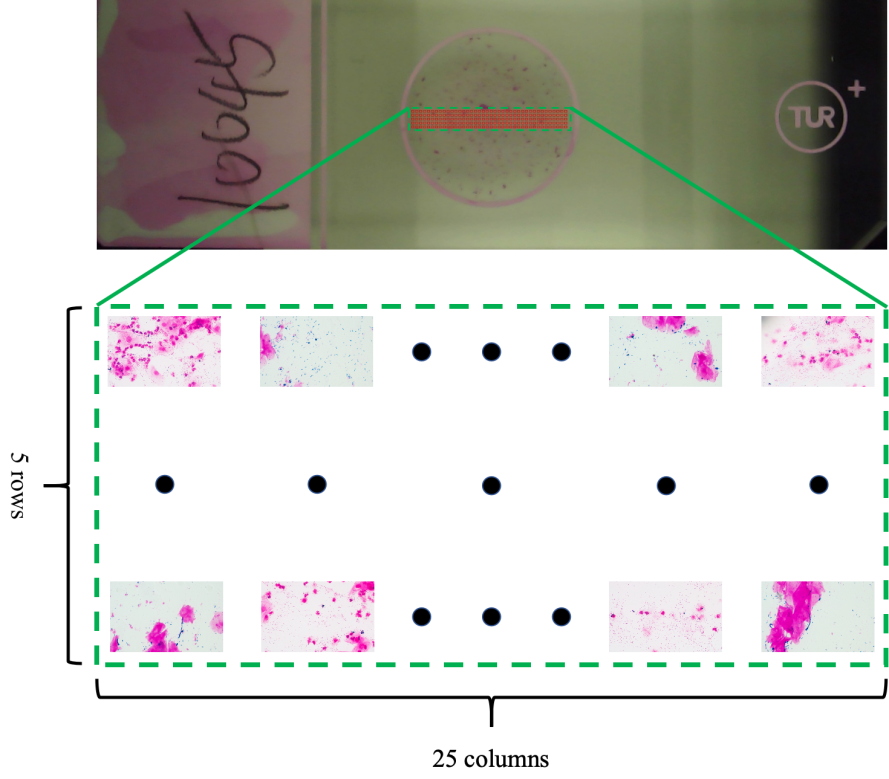


Figure S1. Captured images from one slide. 5 rows × 40 columns = 200 images were captured in each slide, each image covered 226.9 um × 142.5 um actual physical area.


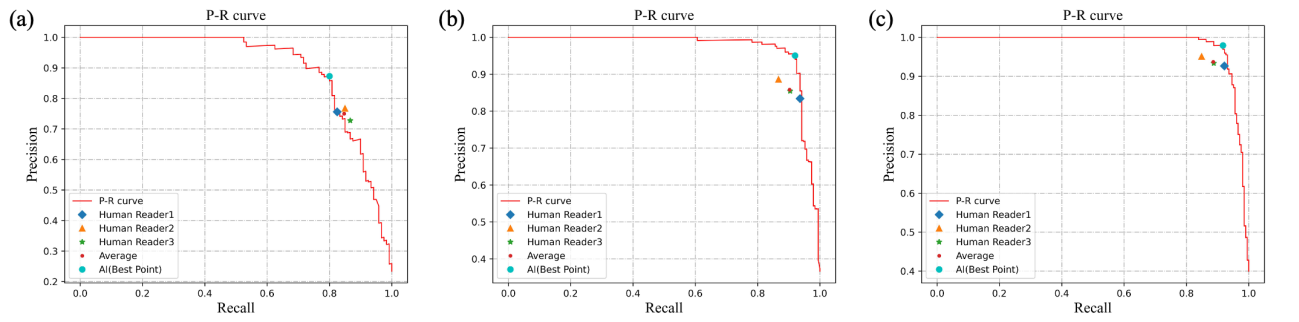


Figure S2. The performance of our cascaded model (P-R curve) and three human practitioners in the slide-level diagnosis. a) was the performance on yeast hyphae diagnosis, b) was the performance on budding yeast diagnosis, c) was the performance on yeast diagnosis. The model demonstrated superior performance compared to the average performance of the three human practitioners in diagnosing budding yeast and yeast. Additionally, the model’s diagnostic performance in yeast hyphae was comparable to the average performance of the three human practitioners.

**Tables**

Tabel S1. The false negative and false positive with AI alone, human alone, and human + AI.

|  | yeast hyphae | | budding yeast | | yeast | | | VVC | |
| --- | --- | --- | --- | --- | --- | --- | --- | --- | --- |
|  | False Negative | False Positive | False  Negative | False Positive | False Negative | False Positive | False Negative | | False Positive |
| AI | 23 | 16 | 15 | 9 | 17 | 4 | 11 | | 16 |
| expert1 | 21 | 32 | 12 | 35 | 16 | 15 | 9 | | 45 |
| expert2 | 18 | 31 | 25 | 21 | 31 | 9 | 6 | | 55 |
| expert3 | 16 | 39 | 18 | 29 | 23 | 13 | 13 | | 41 |
| expert1 + AI | 10 | 9 | 13 | 8 | 4 | 7 | 5 | | 7 |
| expert2 + AI | 16 | 9 | 16 | 6 | 6 | 5 | 10 | | 7 |
| expert3 + AI | 11 | 10 | 8 | 9 | 5 | 6 | 5 | | 8 |
